# Transferability of real-world data across borders for regulatory and health technology assessment decision-making

**DOI:** 10.3389/fmed.2022.1073678

**Published:** 2022-11-16

**Authors:** Ashley Jaksa, Patrick J. Arena, Kelvin K. W. Chan, Rami H. Ben-Joseph, Páll Jónsson, Ulka B. Campbell

**Affiliations:** ^1^Scientific Research and Strategy, Aetion, Inc., New York, NY, United States; ^2^Department of Epidemiology, University of California, Los Angeles, Los Angeles, CA, United States; ^3^Sunnybrook Health Sciences Centre, University of Toronto, Toronto, ON, Canada; ^4^Canadian Centre for Applied Research in Cancer Control, Toronto, ON, Canada; ^5^Big Data Real World Evidence, Jazz Pharmaceuticals, Palo Alto, CA, United States; ^6^National Institute for Health and Care Excellence, Manchester, United Kingdom

**Keywords:** transferability, real-world data, regulatory decision-making, health technology assessments, data relevance

## Abstract

Recently, there has been increased consideration of real-world data (RWD) and real-world evidence (RWE) in regulatory and health technology assessment (HTA) decision-making. Due to challenges in identifying high-quality and relevant RWD sources, researchers and regulatory/HTA bodies may turn to RWD generated in locales outside of the locale of interest (referred to as “transferring RWD”). We therefore performed a review of stakeholder guidance as well as selected case studies to identify themes for researchers to consider when transferring RWD from one jurisdiction to another. Our review highlighted that there is limited consensus on defining decision-grade, transferred RWD; certain stakeholders have issued relevant guidance, but the recommendations are high-level and additional effort is needed to generate comprehensive guidance. Additionally, the case studies revealed that RWD transferability has not been a consistent concern for regulatory/HTA bodies and that more focus has been put on the evaluation of internal validity. To help develop transferability best practices (alongside internal validity best practices), we suggest that researchers address the following considerations in their justification for transferring RWD: treatment pathways, nature of the healthcare system, incidence/prevalence of indication, and patient demographics. We also recommend that RWD transferability should garner more attention as the use of imported RWD could open doors to high-quality data sources and potentially reduce methodological issues that often arise in the use of local RWD; we thus hope this review provides a foundation for further dialogue around the suitability and utility of transferred RWD in the regulatory/HTA decision-making space.

## Introduction

Randomized controlled trials (RCTs) are the primary source of evidence for regulatory and health technology assessment (HTA) decision-making (1). However, there has been increasing consideration of real-world data (RWD) and real-world evidence (RWE) in the regulatory/HTA decision-making process to complement RCTs and address evidence gaps (2–5). For instance, a retrospective analysis of single-arm HTA submissions reported that the proportion of submissions that included RWD-based external control arms (ECAs) increased 22% per year over 2015–2019 (6). In response to the growing use of RWD, there has also been a proliferation of RWE guidelines that provide important recommendations and tools for the proper conduct of RWE studies (7–13). A main focus of these guidelines is ensuring that RWD are “fit-for-purpose,” (9) meaning that the data are high-quality/reliable (i.e., accurate, complete, properly audited, and traceable) and relevant (i.e., contain key data elements, sufficient in sample size, and representative) to the research question (12).

Despite the growth of associated RWE guidelines, regulatory/HTA decision-makers are still concerned that RWD submissions may not meet data fitness standards. For example, a 2022 survey of European HTA agencies noted that some of the most important barriers to using RWE in reimbursement decisions were the lack of “necessary data sources” and the “long time to data access” associated with RWD (5). Similarly, several investigations into the use of RWE in Food and Drug Administration (FDA) submissions concluded that data quality was a frequent concern cited by FDA reviewers in their appraisals (14–16). In order to overcome some of these data fitness problems, investigators may turn to high-quality, accessible data from outside the jurisdiction where a regulatory/HTA decision is to be made (i.e., they may use transferred RWD) (17); in such scenarios, transferred RWD may increase the availability of key data elements and/or sample size, potentially improving the overall relevance.

However, there can be challenges related to the use of transferred RWD, particularly in the context of comparisons with a locally sourced dataset. The patient populations in the local settings may not be comparable to patient populations in the imported data sources, thereby potentially introducing threats to internal validity from confounding and measurement error. Furthermore, the representativeness of imported RWE results may be compromised when such data are used to answer a local question. While similarity in patient populations and generalizability of results are always considerations in RWE studies regardless of the RWD location, these considerations are especially important when considering the transferability of RWD from one jurisdiction to another. Still, researchers in certain regions of the world (18–22) frequently utilize imported RWE due to the significant cost of the resources needed for local RWD generation as well as issues related to small local populations and privacy. Thus, RWD transferability should continue to be a key consideration for regulatory/HTA bodies when assessing RWE studies.

Consequently, concerns from decision-makers may arise when imported RWD sources are used. For instance, an investigation of National Institute for Health and Care Excellence (NICE) technology appraisals of cancer drugs from April 2011 to October 2018 found that there were “several instances” in which NICE questioned the applicability of an identified RWD source to the patient population of interest (23). In order to mitigate such concerns from decision-makers regarding imported RWD, researchers will need to follow guidance on generating valid RWE. However, there currently is a patchwork of guidance on *defining* decision-grade RWE studies and how to use them in decision-making (10); this situation may therefore inadvertently promote the use of poor-quality imported RWD in local regulatory/HTA submissions and/or result in hesitance among researchers to invest in imported RWD sources as their acceptability may be viewed as uncertain. Researchers need clear guidance from regulatory/HTA bodies as well as appropriate tools to ensure that transferred RWD follow best practices and are suitable to the decision at hand. This paper summarizes existing stakeholder guidance on the transferability of international RWD and proposes additional considerations for researchers looking to justify RWD transferability based on themes identified from the stakeholder guidance and selected case studies.

## Overview of existing guidance on the transferability of RWD

We performed a high-level review of transferability recommendations within RWE guidance from North American and European stakeholders based on a methodology described elsewhere (10). From this review, we found that four stakeholders—the Agency for Healthcare Research and Quality (AHRQ) in the United States (US) (24), the US FDA (12, 25), the Institut für Qualität und Wirtschaftlichkeit im Gesundheitswesen (IQWiG) in Germany (26), and NICE in the United Kingdom (UK) (13)—published guidances that explicitly mention the transferability of international RWD (Table 1). More specifically, AHRQ, FDA, IQWiG, and NICE all discussed the importance of assessing the imported RWD fitness in terms of differences in the treatment pathways and/or healthcare system and justifying their use for the local patient context. However, only AHRQ and NICE guidance directly acknowledged that imported data may sometimes be the most suitable option for a robust assessment depending on the context (e.g., when the intervention is only available outside the local geography). Ultimately, our review indicated that there is a degree of consensus among AHRQ, FDA, IQWiG, and NICE on the need to assess and justify the use of imported RWD; however, these recommendations are high-level, and additional effort is needed to operationalize these recommendations and move toward comprehensive guidance.

**Table 1:** Recommendations from key stakeholders on the transferability of RWD/RWE.

**Stakeholder (Country)**	**Year**	**Guidance document**	**Recommendation**
AHRQ (US)	2013	Developing a Protocol for Observational Comparative Effectiveness Research: A User's Guide (24)	AHRQ states that “**a sound justification for selecting a non-US data resource, a solid understanding of the similarities and differences of the non-US versus the US systems, as well as careful discussion of whether the results of the study can be generalized to US populations** will help other researchers and health care practitioners interpret and apply the results.” AHRQ also highlights that the establishment of **national EMR systems in other countries** makes it easier “to obtain **complete, long-term medical records and to follow individuals** in longitudinal studies.”
FDA (US)	2013	Best Practices for Conducting and Reporting Pharmacoepidemiologic Safety Studies Using Electronic Healthcare Data (25)	When using a data source from a country other than the US, FDA recommends that investigator provide **an explanation of how the healthcare system and market availability “might affect the generalizability** of the results to the US population.”
	2021	Real-World Data: Assessing Electronic Health Records and Medical Claims Data To Support Regulatory Decision Making for Drug and Biological Products (12)	For non-US data sources, FDA recommends providing **an explanation of how the healthcare system and prescribing and use practices might affect the generalizability** of the study results to the US population.
IQWiG (Germany)	2020	Development of scientific concepts for the generation of routine practice data and their analysis for the benefit assessment of drugs according to §35a Social Code Book V—rapid report (26)	IQWiG states that “analyses that use **data generated outside of the German healthcare context of interest must justify that these data can be classified as routine practice data in terms of health care in Germany or that deviations are not relevant** for the effect estimate.”
NICE (UK)	2022	NICE real-world evidence framework (13)	NICE states two main points: (1) “**International data is likely to be of particular value when an intervention has been available in another country** before becoming available in the UK or in the context of **rare diseases**,” and (2) “Consideration needs to be given as to how **any differences in the treatment pathways or care settings** seen in the analytical sample and the NHS may impact on the relevance of results. This is especially important when using international data.”

AHRQ, Agency for Healthcare Research and Quality; EMR, electronic medical records; FDA, Food and Drug Administration; IQWiG, Institut für Qualität und Wirtschaftlichkeit im Gesundheitswesen; NHS, National Health Service; NICE, National Institute for Health and Care Excellence; RWD, real-world data; RWE, real-world evidence; UK, United Kingdom; US, United States.

## Key observations from selected transferability case studies

Examples of RWD transferability within regulatory/HTA assessments in the public domain are challenging to identify, as discussions around data fitness typically focus on quality/reliability as well as specific aspects of relevance (e.g., availability of key data elements) and leave the evaluation around transferability to the periphery, if it is even mentioned at all.

When imported RWD are submitted for safety and/or efficacy evaluations, it may not be clear if regulatory agencies consider the transferability of RWD. For example, the FDA required the sponsor of varenicline (CHANTIX^®^/CHAMPIX^®^) to submit a post-marketing safety study to compare pregnancy and birth outcomes among pregnant women exposed to varenicline with women who smoked during pregnancy and with non-smoking pregnant women (27). The sponsor submitted a population-based, prospective cohort study based on registries in Denmark and Sweden, countries that routinely track major life and health events, including pregnancy and birth outcomes (27). The publicly available label update and supplemental approval letter note potential misclassification of the outcomes and exposure but do not comment on the use of data from outside the US (28).

Similarly, it may not be clear that HTA agencies consider RWD transferability when assessing therapies for cost-effectiveness and reimbursement. For instance, NICE assessed atezolizumab (TECENTRIQ^®^) for reimbursement in 2018 for treatment of locally advanced or metastatic non-small cell lung cancer (NSCLC) after chemotherapy (29). To extrapolate overall survival (OS) beyond the RCT results in the cost-effectiveness model, the sponsor supplemented UK-based National Lung Cancer Audit data with US-based RWD from the National Cancer Institute's Surveillance, Epidemiology and End Results and an observational study using Flatiron data. The sponsor used these imported RWD to estimate the percentage of patients that survived for five years in the justification for their OS extrapolation methodology and cost-effectiveness results. While there were documented exchanges between NICE and the sponsor debating the statistical methods of extrapolation, the US-based RWD's transferability to the UK setting was not noted in their public-facing review (29). In this case, NICE accepted the sponsor's extrapolation methods and recommended reimbursement.

In both these cases, the agencies' public documentation was silent on the appropriateness of transferring imported data to the local context. Conversely, an example of an agency's comments on transferability can be seen in the 2021 evaluation of the added benefit of entrectinib (ROZLYTREK^®^) as a monotherapy for the treatment of adult patients with *ROS1*-positive advanced NSCLC over available therapies by Gemeinsamer Bundesausschuss (G-BA) (30). As part of this evaluation, the sponsor submitted an ECA generated from US-based RWD to compare entrectinib to the appropriate comparator therapy, crizotinib, on OS. G-BA critiqued the ECA due to the lack of randomization and the potential for systematic bias, which the G-BA deemed to be “evident” based on the substantial differences in OS between the ECA and a pivotal crizotinib RCT. In addition, G-BA noted that the “transferability of the data [from the US] to the German healthcare context [was] questionable because of structural differences in the health care systems” (30). The potential of systematic bias, from a lack of randomization, to account for the effect size between the single-arm study and the ECA ultimately led G-BA to not consider the ECA as supportive evidence.

These case studies illustrate that RWD transferability is usually not highlighted in the publicly available documentation as a key consideration. The limited guidance from regulatory/HTA bodies might have contributed to or reflect the variable consideration of transferability among these case studies. Similarly, the silence on RWD transferability could be the result of how the RWD were used in the decision-making (i.e., primary vs. secondary evidence) and what other evidence was available in addition to the transferred RWD. Certainly, it may be the case that certain aspects of transferability were discussed by these agencies in private but not in their published evaluations, thereby limiting our ability to directly infer their importance. As a result, we argue that RWD transferability should garner more attention and be more explicitly appraised in regulatory/HTA evaluations since the use of imported RWD could open the doors to high-quality data sources and potentially reduce methodological issues that often arise in the use of local RWD. More detailed guidance would help ensure that the use of imported RWD is justified for the research question of interest.

## Considerations for the transferability of imported RWD

While tools exist to help researchers identify and select fit-for-purpose RWD (9), there are additional factors to consider when using RWD outside the jurisdiction of interest. To further refine and operationalize the current guidelines around RWD transferability and to help develop best practices, we suggest that researchers address the following considerations in their justification for data selection: treatment pathways, the healthcare system, incidence/prevalence of indication, and patient demographics (Table 2).

**Table 2:** Considerations for researchers to keep in mind for the transferability of RWD.

**Category**	**Considerations**
Treatment patterns	Clinical guidelines Current standards of care Prescribing patterns Availability of therapies
Healthcare system	Location of care Access to care Cost of care
Prevalence/incidence of indication	Heterogeneity across geographies Confounding factors
Patient demographics	Age Sex Race and ethnicity Socioeconomic status Genetic markers

RWD, real-world data.

### Treatment pathways

AHRQ, FDA, IQWiG, and NICE noted that in order to evaluate the transferability of international RWD, treatment pathways should be similar between jurisdictions and any differences should be addressed. Potential impacts on the results of the study should therefore be described. Treatment pathways can encompass everything from evidence-based recommendations in clinical guidelines to actual prescribing patterns and the availability of therapies, all of which can vary by location. For instance, standard of care (e.g., gastric cancer treatments) (31, 32), prescribing patterns (e.g., opioid prescriptions) (33, 34), and the availability of therapies (35–37) may all be different depending on the country context. If there are discrepancies in how patients are treated between where the RWD are collected and where the sponsor is trying to apply the study findings, these differences may limit the internal validity and/or the representativeness of the RWE study results. When these discrepancies become too large, inferences from the transferred data to the population of interest may be problematic.

### Healthcare system

Similarly, AHRQ, FDA, IQWiG, and NICE agreed that the healthcare systems should be similar between jurisdictions if RWD are to be considered transferable. We therefore suggest that researchers focus on documenting similarities in patient access to care and where care is delivered. Researchers should also be particularly aware of potential challenges associated with cost of care and how that affects access; issues with access may be most prominent when transporting US-based RWD to a non-US setting, as high uninsurance rates and cost-sharing can result in diminished levels of healthcare access in the US (38). Likewise, differences in how the healthcare systems function across countries are also important to keep in mind; for instance, certain countries (e.g., Canada) have some form of a universal healthcare system while others (e.g., the US) rely on a mixture of private insurance and government programs (39). As seen with G-BA's entrectinib evaluation (30), such “structural differences” between healthcare systems can limit the applicability of the RWE. Since it is not possible to easily account for health system “structural differences” in the analytic phase of the study (unlike an observed confounder), it is critical to avoid the transfer of RWD when there is a substantial mismatch of healthcare systems between the RWD source and the target jurisdiction; this mismatch can become especially problematic when it may have a significant impact on certain study aspects (e.g., outcome ascertainment) that are necessary for valid inference. In cost-effectiveness studies, RWD transferability may also be limited since costs are normally unique to the local healthcare system and thus present another potential instance of mismatch between healthcare systems.

### Incidence/prevalence of indication

An additional consideration beyond the current recommendations from AHRQ, FDA, IQWiG, and NICE concerns differences in the incidence/prevalence of indication. Due to different population mixes and environmental factors, the incidence/prevalence of an indication can be heterogeneous across geographies (40, 41). This situation can actually be beneficial to the researcher though if it allows them to use RWD from another jurisdiction with a higher incidence/prevalence to increase the sample size/power of the study. However, it is essential to understand why differences in incidence/prevalence exist between the geographies. Factors associated with the increased incidence/prevalence in one geography can lead to a decrease in the representativeness of the RWE findings in other geographies. If the transferred RWD are being used as a control for locally sourced data, such factors can also hamper internal validity when they are unobserved/unmeasured or otherwise uncontrollable (e.g., through a positivity violation) in the analysis. As seen with the varenicline post-marketing safety study using Scandinavian data, the prevalence of major congenital malformations observed in the Scandinavian cohorts was similar to the background level in the US (28), thus providing some evidence of transferability.

### Patient demographics

Patient demographics, such as age, sex, race/ethnicity, socioeconomic status, and genetic profile, are all common risk factors for disease and can thus impact the incidence/severity of the indication as well as health outcomes. These characteristics can vary widely across geographies; for example, even within one country, like the US, patient demographics differ substantially by state (42, 43). While it is unrealistic that RWD transferability hinges on an exact match in patient demographics between geographies, it is important to consider those patient characteristics that are highly correlated with the outcomes that are being measured in the RWE study. If differences exist, the researcher should explore how these discrepancies impact the representativeness of the findings (e.g., through subgroup analysis based on these demographics). This recommendation is especially salient for indications that differentially affect certain demographic groups, such as sickle cell anemia (44), breast cancer (45), and HIV/AIDS (46).

## Discussion

The generation of high-quality RWE for regulatory/HTA decision-making relies on the availability of comprehensive guidance and tools that enable researchers to identify fit-for-purpose RWD. Use of RWD from outside the jurisdiction of interest is one way that researchers can meet data fitness standards, possibly reduce methodological concerns, and increase efficiency by conducting studies to support multi-jurisdictional decision-making. When considering data from other locations, a fitness assessment should therefore always include transferability considerations that seek to create an optimal balance between representativeness and availability of key data elements/sample size sufficiency in order to identify relevant RWD.

Stakeholders are aware of the need to use imported RWD and have issued high-level guidance on considerations for international RWD transferability (12, 13, 24–26). This paper has attempted to further refine these transferability recommendations, but additional work is needed to define minimal criteria necessary for RWD transferability and to develop processes that researchers can follow to ensure they are meeting decision-makers' expectations. Alternatively, if regulators and HTA agencies are hesitant to define minimal criteria, emphasis should be placed on the need for transparency and clear dialogue when utilizing imported RWD sources. In the interim, demonstration projects are a useful way to explore when imported RWD are transferable and when they are not. For example, replicating RWE studies in various geographies that have measurable differences in treatment patterns, healthcare systems, the prevalence/incidence of indications, and/or patient demographics can isolate the effect of these factors on the final results.

Lastly, we acknowledge that this paper focuses on the considerations for RWD transferability but does not delve into the contextual factors that allow the subsequent RWD to be deemed as useful RWE by decision-makers. For example, deliberations on the nuances between study types (e.g., the relative importance of local data for a natural history study vs. a comparative effectiveness assessment) or how the RWE will be used in the submission (e.g., primary vs. complementary evidence) have not been discussed. However, our review of the stakeholder guidance and the selected case studies revealed that gaps exist in the operationalization of the basic RWD characteristics needed to justify data source selection; thus, we believe that our focus specifically on data considerations is warranted because it provides a framework for the selection of valid RWD, which is a necessary first step in the generation of suitable RWE. Ultimately, as RWE is utilized more frequently by researchers and decision-makers, the issues specific to the transferability of imported RWD will become increasingly more central to the evaluation of study quality. We hope that this paper therefore furthers the dialogue around the place of RWD transferability in the regulatory/HTA decision-making landscape.

## Data availability statement

The stakeholder guidance and selected case studies that support the findings of this perspective are available from the corresponding author upon reasonable request.

## Author contributions

AJ, KC, RB-J, and PJ conceived the original study concept. AJ and PA performed the stakeholder guidance and case selection review with input from UC. AJ and PA drafted the manuscript with input from KC, RB-J, PJ, and UC. All authors read and approved the final manuscript.

## Conflict of interest

Author AJ is an employee of and has ownership stake in Aetion, Inc. Author PA is a current consultant for Aetion, Inc., and former contractor for Pfizer, Inc. Author RB-J was an employee of and holds stock options in Jazz Pharmaceuticals. Author UC is an employee of and has ownership stake in Aetion, Inc.; holds stock options in Pfizer, Inc. Authors PJ and KC have no competing interests to declare.

## Publisher's note

All claims expressed in this article are solely those of the authors and do not necessarily represent those of their affiliated organizations, or those of the publisher, the editors and the reviewers. Any product that may be evaluated in this article, or claim that may be made by its manufacturer, is not guaranteed or endorsed by the publisher.
